# Roles of XB130, a novel adaptor protein, in cancer

**DOI:** 10.1186/2043-9113-1-10

**Published:** 2011-03-17

**Authors:** Atsushi Shiozaki, Mingyao Liu

**Affiliations:** 1 Division of Digestive Surgery, Department of Surgery, Kyoto Prefectural University of Medicine, Kyoto, 602-8566, Japan; 2 Latner Thoracic Surgery Research Laboratories, University Health Network Toronto General Research Institute, Toronto, Ontario, M5G 1L7, Canada; 3 Department of Surgery, Faculty of Medicine, University of Toronto, Toronto, Ontario, M5G 2C4, Canada

## Abstract

Adaptor proteins, with multi-modular structures, can participate in the regulation of various cellular functions. During molecular cloning process of actin filament associated protein, we have discovered a novel adaptor protein, referred to as XB130. The human *xb130 *gene is localized on chromosome 10q25.3, and encodes an 818 amino acid protein. The N-terminal region of XB130 includes several tyrosine phosphorylation sites and a proline-rich sequence that might interact with Src homology 2 and 3 domain-containing proteins, respectively. Our studies have indeed implicated XB130 as a likely substrate and regulator of tyrosine kinase-mediated signaling. Down-regulation of endogenous XB130 with small interfering RNA reduced c-Src activity, IL-8 production and phosphorylation of Akt in human lung epithelial cells. Further, XB130 binds the p85α subunit of phosphatidyl-inositol-3-kinase and subsequently mediates signaling through RET/PTC in thyroid cancer cells. Knockdown of XB130 using small interfering RNA inhibited G_1_-S phase progression, induced spontaneous apoptosis and enhanced intrinsic and extrinsic apoptotic stimulus-induced cell death in human lung and thyroid cancer cells. Growth of tumors in nude mice formed from XB130 short hairpin RNA stably transfected human thyroid cancer cells were significantly reduced, with decreased cell proliferation and increased apoptosis. Further, XB130 has a high affinity to lamellipodial F-actin meshwork and is involved in the motility and invasiveness of cancer cells. Gene expression profiling identified 246 genes significantly changed in XB130 short hairpin RNA transfected thyroid cancer cells. Among them, 57 genes are related to cell proliferation or survival, including many transcription regulators. Pathway analysis showed that the top ranked disease related to XB130 is Cancer, and the top molecular and cellular functions are Cellular Growth and Proliferation, and Cell Cycle. These observations suggest that the expression of XB130 may affect cell proliferation, survival, motility and invasion in various cancer cells. A deeper understanding of these mechanisms may lead to the discovery of XB130 as an important mediator in tumor development and as a novel therapeutic target for cancer.

## Review

### Introduction

Adaptor proteins are molecules of modular structures without enzymatic activity, composed of multiple protein-protein and/or protein-lipid interacting domains, through which they link signaling components to form macromolecular complexes and propagate cellular signals [1,2]. Depending on the functional role of the interacting partner and the specific biological event that is triggered by these interactions, adaptor proteins can participate in the regulation of different signaling pathways. A good example of how adaptor proteins are involved in signal transduction is the activation of c-Src protein-tyrosine kinases by adaptor proteins via protein-protein interactions. Adaptor proteins are also important to mediate signals initiated via receptor-tyrosine kinases in responses to extracellular stimuli [3,4], and together with non-receptor protein-tyrosine kinases to orchestrate the signal transduction elicited by either ligand receptor interactions or by cellular structure reorganization [5]. Further, a number of adaptor proteins have been demonstrated to regulate tumorigenesis. For example, actin filament associated protein (AFAP) is required for actin stress fiber formation and cell adhesion, and is critical for tumorigenic growth in prostate cancer [6,7]. Tyrosine kinase substrate 5 is a scaffolding adaptor protein with five Src homology (SH) 3 domains, co-localizes to podosomes and regulates migration and invasion of different human cancer cells [8,9]. These findings support a broader investigation of adaptor proteins on tumorigenesis and their potentiality as diagnostic biomarkers and therapeutic targets of cancer.

During our studies aimed at the characterization of the AFAP [10-12], we cloned a novel 130 kDa protein, referred to as XB130 [13]. Our studies have indeed indicated that XB130 plays, as an adaptor, important roles in the regulation of signal transduction, cell proliferation, survival, motility and invasion [13-16]. In this review, we focus on studies relate to both XB130 and cancer progression.

### Molecular structure of XB130

**Figure 1:**
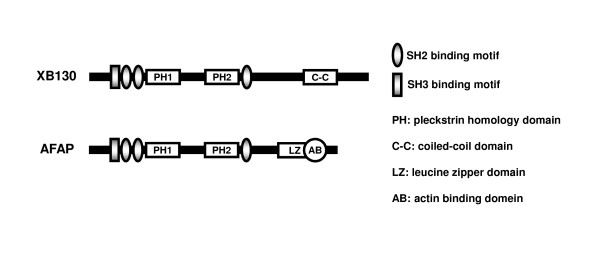
**Schematic representation of the XB130 and AFAP protein structures**. The N-terminal region of XB130 includes several tyrosine phosphorylation sites and a proline-rich sequence that may interact with Src homology (SH) 2 and SH3 domain-containing proteins. The middle portion harbors two pleckstrin homology (PH) domains, while the C-terminal region contains a coiled-coil domain. A common feature of XB130 (818aa) and AFAP (730aa) is the presence of potential SH2, SH3-binding sites and two PH domains. A coiled-coil domain of XB130 shares partial similarity with the leucine zipper domain and in AFAP.

The human *xb130 *gene is localized on chromosome 10q25.3 and encodes 818 amino acids with an apparent molecular size of approximately 130 kDa [13]. As an adaptor protein, the overall structure of XB130 shares similarity with AFAP, thus it is also known as actin filament associated protein 1-like 2 (AFAP1L2). The N-terminal region of XB130 includes several tyrosine phosphorylation sites and a proline-rich sequence which can potentially interact with SH2 and SH3 domain-containing proteins, respectively (Figure 1) [13,14]. The middle portion harbors two pleckstrin-homology (PH) domains that may target proteins to cellular membranes through interactions with specific phospholipids, such as phosphoatidylinositol-3, 4, 5-triphosphate. The C-terminal region contains a coiled-coil domain, which might be involved in protein oligomerization and DNA binding. A common feature of XB130 and AFAP is the presence of a proline-rich motif, several potential SH2-binding sites and two PH domains (Figure 1) [13,14]. A coiled-coil domain of XB130 shares partial similarity with the leucine zipper domain in AFAP. Despite these similarities, XB130 does not behave like an actin filament-associated protein. The actin-binding site that is present in the C terminus of AFAP [17] is only partially present in XB130. The distribution of AFAP appears to be along the stress fiber, and through its interaction, AFAP transmits physical force and mediates mechanical stretch-induced c-Src activation [12,18]. On the other hand, the diffuse distribution of XB130 in the cytoplasm suggests that XB130 plays a different role in signal transduction and cellular functions [13]. XB130's tissue distribution was determined by using northern blot analysis and high expression of XB130 was found in human thyroid and spleen [14].

### Regulation of tyrosine kinase-mediated signaling by XB130

Our studies have implicated XB130 as a likely substrate and regulator of tyrosine kinase-mediated signaling [13,14]. Endogenous XB130 interacts with c-Src tyrosine kinase [13]. Their co-expression in COS-7 cells resulted in activation of c-Src and elevated tyrosine phosphorylation of multiple proteins, including XB130 itself. XB130 expression in HEK293 cells enhanced serum response element- and AP-1-dependent transcriptional activation mediated by c-Src. Down-regulation of endogenous XB130 with small interfering RNA (siRNA) reduced c-Src activity, IL-8 production, epidermal growth factor (EGF)-induced phosphorylation of Akt and GSK3β in human lung cancer A549 cells [13].

Further, our studies revealed expression of XB130 in human thyroid tissue, and we found that XB130 is a downstream mediator of the signaling cascade propagated by RET/PTC, a genetically rearranged, constitutively active, thyroid cancer-specific tyrosine kinase [14]. RET/PTC plays a pathogenic role and exhibits transforming ability by exerting its effects on differentiation, mitogenic and metastatic potential in papillary thyroid cancer [19,20]. XB130 couples RET/PTC signaling to the phosphatidyl-inositol-3-kinase (PI3K)/Akt signaling through a specific binding site to p85α subunit of PI3K [14]. A study investigating the implications of Src tyrosine kinases in certain colorectal cancer by Emaduddin et al. identified XB130 from SW629 colorectal cancer cells, as one of the tyrosine phosphorylated proteins binding to Lck-SH2 domain [21]. Lck, is a Src family member that is not detectable in normal colonic epithelium, but becomes aberrantly expressed in a subset of colorectal carcinomas. These findings indicate that XB130 has an important role in the regulation of tyrosine kinase-mediated signaling.

### Roles of XB130 in cell cycle and survival

**Figure 2:**
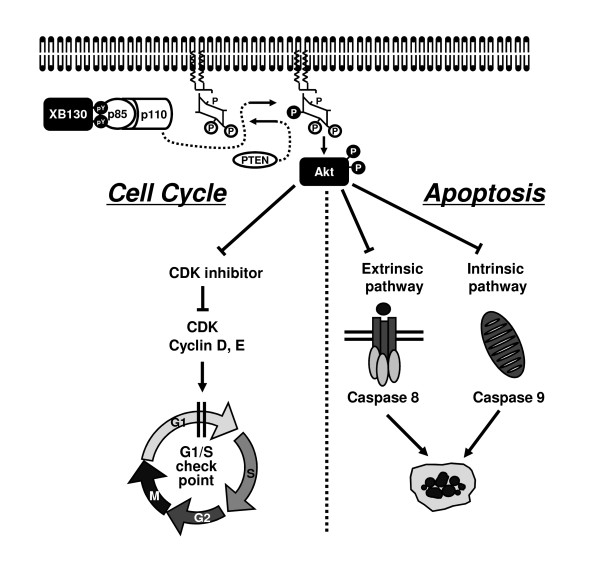
**Roles of XB130 in cell cycle and survival of cancer**. XB130 specifically binds p85α subunit of PI3K, which subsequently activate Akt. Akt plays an essential role in cell proliferation and survival.

To investigate the role of XB130 in cancer cell cycle progression, we conducted knockdown experiments with XB130 siRNA [13-15]. Down regulation of XB130 reduced cell cycle progression from G_1 _to S phase in human lung cancer cell line, A549 and human thyroid cancer cell lines, TPC1 and WRO (Figure 2) [13-15]. The expression of cell proliferation markers, Ki-67 and PCNA, were also reduced in XB130 siRNA treated WRO cells [15]. Down-regulation of XB130 induced apoptosis and enhanced extrinsic or intrinsic apoptotic stimulus-induced early and late apoptosis in WRO cells (Figure 2) [15]. In TPC1 cells, down-regulation of XB130 accelerates the apoptotic process [14]. Further, to determine the roles of XB130 *in vivo*, we established XB130 short hairpin RNA (shRNA) stably transfected WRO cell lines and used a xenograft model in nude mice [15]. Growth of tumors in nude mice formed from XB130 shRNA stably transfected WRO cells were significantly reduced, with decreased cell proliferation and increased apoptosis. These findings indicate that XB130 expression levels affected cell proliferation and survival in cancer cells (Figure 2).

### Roles of XB130 in cell motility and invasion

We further found that XB130 has a high affinity to lamellipodial F-actin meshwork and is involved in the motility and invasiveness of tumor cells. XB130 exhibited robust translocation to the cell periphery in response to various stimuli (including EGF, wounding and expression of constitutively active Rac) that elicit lamellipodium formation [16]. Structure-function analysis revealed that both the XB130 N-terminus and C-terminus harbor critical regions for its translocation to lamellipodia [16]. In TPC1 thyroid papillary carcinoma cells, silencing endogenous XB130 decreased the rate of wound closure, inhibited cell invasion through Matrigel, reduced lamellipodial persistence and slowed down spreading [16]. Thus, XB130 is a novel Rac/cytoskeleton-regulated and cytoskeleton-regulating adaptor protein, which exhibits high affinity to lamellipodial F-actin and impacts motility and invasiveness of tumor cells.

### Gene expression profile in XB130 shRNA transfected cells

To determine the molecular mechanisms by which XB130 regulates cellular functions, we analyzed gene expression profiles in XB130 shRNA transfected cells by microarray and bioinformatics studies [15]. Microarray analysis identified 246 genes significantly changed in XB130 shRNA transfected cells. Among them, 57 genes, such as HSPA1A, BHLHE40, TOB1, DDIT3, SLC7A11 and MYC are related to cell proliferation or survival, including many transcription regulators. Ingenuity Pathway Analysis showed that the top ranked disease related to XB130 is Cancer, and the top molecular and cellular functions are Cellular Growth and Proliferation, and Cell Cycle [15]. These results indicate that the expression level of XB130 influences genes related to cellular growth and proliferation, cell cycle, cell death and organismal survival. Furthermore, Cunha et al. performed gene expression profiling using 102 soft tissue tumor samples, and found XB130 as one of the genes highly related to local aggressiveness [22]. Therefore, in addition to thyroid cancer, XB130 may also play important roles in other neoplasms.

## Conclusions

**Figure 3:**
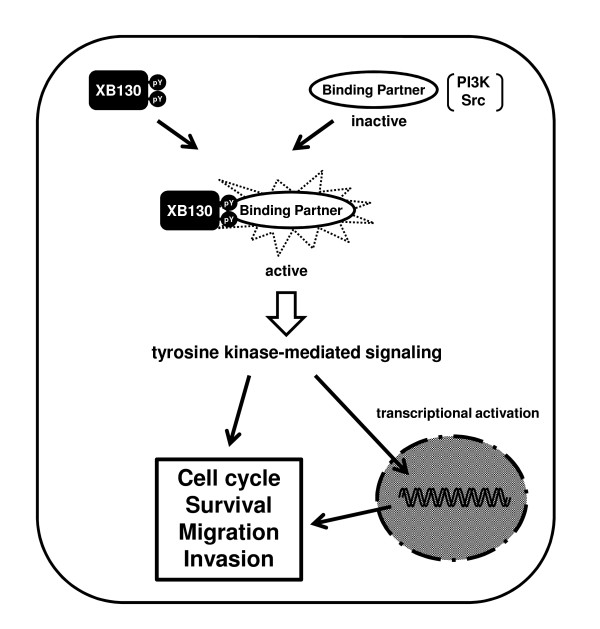
**Roles of XB130 in cancer behavior**. XB130 interacts with binding partners and regulates cell cycle, survival, migration and invasion of cancer through tyrosine kinase-mediated signaling.

We have provided evidence that XB130 plays important roles in tumor progression by promoting cell proliferation, survival, motility and invasion in various cancer cells (Figure 3). XB130 has profound effects on expression of genes related to tumorigenesis. These findings suggest that XB130 could be a novel oncoprotein in cancer. A deeper understanding of these mechanisms may lead to the discovery of XB130 as an important mediator in tumor development and as a novel therapeutic target for cancer.

## Lists of abbreviations

AFAP: actin filament associated protein; AFAP1L2: actin filament associated protein 1-like 2; PH domain: pleckstrin-homology domain; PI3K: phosphatidyl-inositol-3-kinase; SH domain: Src homology domain; shRNA: short hairpin RNA; siRNA: small interfering RNA

## Competing interests

The authors declare that they have no competing interests.

## Authors' contributions

AS carried out experiments concerning this review and wrote this manuscript. ML designed experiments and supervised research. All authors read and approved the final draft.
